# Ontogeny of the Digestive System of Atlantic Salmon (*Salmo salar* L.) and Effects of Soybean Meal from Start-Feeding

**DOI:** 10.1371/journal.pone.0124179

**Published:** 2015-04-29

**Authors:** Christian Sahlmann, Jinni Gu, Trond M. Kortner, Ingrid Lein, Åshild Krogdahl, Anne Marie Bakke

**Affiliations:** 1 Department of Basic Sciences and Aquatic Medicine, Faculty of Veterinary Medicine and Biosciences, Norwegian University of Life Sciences, Oslo, Norway; 2 Nofima AS, Sunndalsøra, Norway; University of Nordland, NORWAY

## Abstract

Despite a long history of rearing Atlantic salmon in hatcheries in Norway, knowledge of molecular and physiological aspects of juvenile development is still limited. To facilitate introduction of alternative feed ingredients and feed additives during early phases, increased knowledge regarding the ontogeny of the digestive apparatus in salmon is needed. In this study, we characterized the development of the gastrointestinal tract and accessory digestive organs for five months following hatch by using histological, biochemical and molecular methods. Furthermore, the effects of a diet containing 16.7% soybean meal (SBM) introduced at start-feeding were investigated, as compared to a fishmeal based control diet. Salmon yolk sac alevins and fry were sampled at 18 time points from hatch until 144 days post hatch (dph). Histomorphological development was investigated at 7, 27, 46, 54 and 144 dph. Ontogenetic expression patterns of genes encoding key digestive enzymes, nutrient transporters, gastrointestinal peptide hormones and T-cell markers were analyzed from 13 time points by qPCR. At 7 dph, the digestive system of Atlantic salmon alevins was morphologically distinct with an early stomach, liver, pancreas, anterior and posterior intestine. About one week before the yolk sac was internalized and exogenous feeding was started, gastric glands and developing pyloric caeca were observed, which coincided with an increase in gene expression of gastric and pancreatic enzymes and nutrient transporters. Thus, the observed organs seemed ready to digest external feed well before the yolk sac was absorbed into the abdominal cavity. In contrast to post-smolt Atlantic salmon, inclusion of SBM did not induce intestinal inflammation in the juveniles. This indicates that SBM can be used in compound feeds for salmon fry from start-feeding to at least 144 dph and/or 4-5 g body weight.

## Introduction

Finding optimal dietary nutrient composition and securing adequate feed intake during early life stages are demanding research tasks in production of many aquatic species. Rearing Atlantic salmon, however, has been relatively unproblematic, at least partially because salmon alevins are relatively well developed at the time of hatch and readily accept dry compound feed from start-feeding. Consequently, little research has been conducted on the functional development of the digestive system of salmon. Most studies on early stages of salmonid development have described effects of water temperature on the timing of hatch, growth, skeletal development, disease susceptibility and yolk utilization [1–4]. Other studies have concerned the effects of toxins on developing salmon [5,6]. A morphological description of the general development of Atlantic salmon from fertilization until complete yolk sac absorption has been published [7], but only limited information on the ontogeny of the gastrointestinal (GI) tract is available.

In recent years, research efforts have focused on finding alternative protein sources in formulated feeds for salmon cultivation as fishmeal availability is limited and market prices have increased [8]. Protein from alternative sources, such as various crops, has replaced fishmeal at increasing proportions. An extensive body of literature has described effects of plant-based protein sources on the growth, digestion and health in post-smolt Atlantic salmon. The use of certain plant ingredients, most notably fullfat and de-fatted (extracted) soybean meal (SBM), is still limited for at least some fish species as endogenous antinutritional factors, toxins and/or antigens challenge fish health and welfare [9]. But only limited information is available on effects of plant ingredients and their antinutrients on juvenile stages of salmon. Indications exist that pre-smolt salmon may be able to tolerate SBM [10] at levels that are known to cause inflammation in the distal intestine of seawater-adapted salmon [11–14]. However, to assess whether alternative protein sources can be safely used in start-feeding diets for salmonids, more focused investigations are needed to increase knowledge on how they may affect functional development and health of the digestive system.

Accordingly, the aim of this study was to increase the basic knowledge on the ontogeny of the digestive system in Atlantic salmon and the possible modulating effects of a plant ingredient in the diet, specifically SBM, chosen due to its potential to induce a diet-related intestinal inflammation with accompanying dysfunction. The development of the GI tract was characterized from hatch through start-feeding until approximately three months post start-feeding—144 days post hatch (dph), equivalent to 1728 day degrees. The effects of a diet containing 16.7% extracted SBM introduced at start-feeding were investigated and compared to a fishmeal-based control diet. The morphological development of the GI tract was evaluated by a detailed histological examination. Quantitative PCR was used for gene expression profiling of key digestive enzymes (pepsinogen; *pep*, trypsinogen 1a; *trp-ia*, bile salt-activated lipase; *bal*, alpha-amylase; *amy*), gastrointestinal peptide hormones (ghrelin, *ghrl*; cholecystokinin, *cck-l*; peptide yy, *pyy*), nutrient and water transporters (peptide transporter, *pept*; sodium dependent glucose transporter, *sglt1*; cluster of differentiation 36, *cd36*; niemann-pick c1-like 1, *npc1l1*; ATP binding cassette g5, *abcg5*; aquaporin 8ab, *aqp8ab*) and T-cell markers (interleukin 17a, *il-17a*; T-cell receptor gamma, *tcrγ)*. Bile salt levels and enzyme activity of trypsin and the brush border enzymes leucine aminopeptidase (LAP) and maltase were also monitored during development. The results of this work provide new insights into the ontogeny of digestive functions in Atlantic salmon, and may aid in assessing the effects of alternative feedstuffs on smolt quality.

## Materials and Methods

### Ethics statement

Rearing of the fish were conducted at Nofima’s Research Station (Sunndalsøra, Norway), which is an approved research facility by Norwegian Animal Research Authority (NARA) and operates in accordance with the Norwegian Regulations of 17 June 2008 No. 822: Regulations relating to Operation of Aquaculture Establishments (Aquaculture Operation Regulations). Up to sacrifice and sampling, the fish were treated as production fish in accordance with aforementioned Aquaculture Operation Regulations. Prior to sampling, randomly chosen, individual fish were removed from their respective tanks with nets at the various time points from 7 to 144 dph (see below), and humanely anaesthetized and euthanized in water containing a lethal dose of anaesthetic, in accordance with the Norwegian Animal Welfare act. No surgical manipulation of live fish was conducted and tissue samples were only retrieved from euthanized fish. The experimental diets contained ingredients commonly used in commercial feeds and do not cause the fish any apparent distress. Hence, no NARA approval was required according to §2 of the Norwegian Regulation on Animal Experimentation.

### Experimental setup and sampling

Disinfected eggs from Atlantic salmon of SalmoBreed origin were purchased from Bolaks A/S (Eikelandsosen, Norway) and hatched at the research station of Nofima AS (Sunndalsøra, Norway). After hatch, alevins were transferred to six tanks with a density of approximately 1200 alevins/tank. The tanks were 60 cm in diameter and supplied with filtered fresh water. The water level was kept at 19 cm at initiation of the feeding period and later elevated to 32 cm when the fish started to swim upwards in the water column. Eggs and alevins were kept at 7–8°C with continuous light until start-feeding. Start-feeding was initiated after the yolk sac had been absorbed at 46 dph. The water temperature from start-feeding was kept at 11–12°C and light was provided continuously.

Feeds were prepared by Nofima AS in Bergen, Norway. One set of triplicate tanks received a fishmeal-based diet (FM), while the second set received a diet containing 16.7% non-GM, hexane-extracted (defatted) SBM obtained from Denofa AS (Fredrikstad, Norway; see Table 1 for diet formulations). The diets were balanced regarding vitamins and minerals according to estimated requirements [15] and formulated to have an approximate protein-to-energy ratio of 25 g/MJ (Table 1). Both diets were extruded, crumbled and sieved into three particle sizes: 0.6 mm, 0.9 mm and 1.3 mm. The feed pellet size given at start-feeding was 0.6 mm, and was subsequently changed to 0.9 mm and 1.3 mm as the fish grew to 1 and 3 g, respectively. The fish were fed continuously by automatic feeders at 10 min intervals with an excess of 20% of the estimated feed requirement. Due to the small size of the feed pellets, feed intake could not be accurately measured.

**Table 1:** Formulation and proximate composition (as fed basis) of the experimental diets.

	FM	SBM
**Ingredient (g/kg)**		
Fishmeal (58/09)^a^	706	564
Extracted SBM (239/08)^b^	-	167
Maize	200	167
NorSalmOil^c^	70	80
Vitamin mix^d^	20	19
Mineral mix^e^	4	4
Carophyll Pink 10%	0.2	0.2
**Proximate composition (g/kg)**		
Dry matter	947	942
Crude protein	519	485
Crude lipid	164	154
Carbohydrates	170	218
Gross energy (MJ/kg)^f^	21.6	21.3
Protein (g)/energy (MJ)	24	23

^a^Norseco-LT, Norsildmel, Bergen, Norway

^b^Extracted Soybean meal, Denofa As, Fredrikstad, Norway

^c^NorSalmOil, Norsildmel, Bergen, Norway

^d^Normin AS, Hønefoss, Norway. Diets supplied with following vitamins per kg diet: vitamin D3, 3000 I.E; vitamin E (Rovimix, 50%), 160mg; thiamine, 20mg; riboflavin, 30mg; pyridoxine-HCl, 25mg; vitamin C (Riboflavin Stay C 35%), 200mg; calcium pantothenate, 60mg; biotin, 1mg; folic acid, 10mg; niacin, 200mg; vitamin B12, 0.05 mg; menadione bisulphate, 20mg.

^e^Normin AS, Hønefoss, Norway. Diets supplied with following minerals per kg diet: magnesium, 750mg; potassium, 800mg; zinc, 120mg; iron, 60 mg; manganese, 30mg; copper, 6mg and selenium; 0.3mg.

^f^Gross energy was calculated using the energy concentrations of 39.5 for lipid, 23.6 for protein, and 17.2 kJ/g for carbohydrates (carbohydrate levels in diets were calculated as: 100 –[water + crude protein + crude lipid + ash]).

Fish were sampled at 18 time points between 7 and 144 dph. Randomly selected fish were euthanized by a lethal dose of tricaine methane-sulfonate (MS222; Argent Chemical Laboratories Inc., Redmont, WA, USA) and rinsed in distilled water before further processing. Body weight and fork length were measured for a representative sample of at least 30 fish per tank at each sampling. For histology, whole fish were fixed in a 4% phosphate-buffered formaldehyde solution for 24h and subsequently transferred to 70% alcohol and stored at 4°C until further processing. For RNA extraction, digestive enzyme activities and bile salt levels, whole fish or isolated tissues were snap-frozen in liquid nitrogen and subsequently stored at -80°C.

To reduce costs, the fish from each of the two diet groups were gathered into one tank per diet following the 96 dph sampling. The fish were otherwise held under the same conditions as described above and continued on the same respective diets until the final sampling at 144 dph.

### Histology

Formalin-fixed whole fish, randomly selected at days 7, 27, 46, 54 (n = 3 per time point and tank), and 144 (n = 12 per diet) dph were routinely dehydrated in ethanol, equilibrated in xylene and embedded in paraffin according to standard histological procedures. The fish were cut into serial sagittal sections of 3–5 μm and stained with haematoxylin and eosin (H&E). The sections were evaluated under a light microscope (Carl Zeiss, Inc. UK) and representative images were taken. The images were color-adjusted and structures labelled using Adobe Photoshop Elements 7.0.

### Gene expression

For RNA extraction, whole alevins sampled at 7, 17, 27 and 38 dph were prepared by removing heads and tails, while from 46, 49, 54, 60, 67, 74, 81, 96 and 144 dph, the gastrointestinal tract was excised. All preparations were performed on ice and tissues were immediately placed in ice cold Trizol (Life Technologies, Carlsbad, CA, USA). Bodies/tissues from three fish per tank were pooled (n = 3 pooled samples, *i*.*e*. 9 individual fish per time point and diet) and homogenized on ice using an Ultra Turrax (IKA Werke, Germany). The homogenate was centrifuged at 12,000 x *g* for 5 min and the supernatant was transferred to a Direct-zol column (Zymo Research, Irvine, CA, USA). Total RNA was extracted using a Direct-zol RNA MiniPrep kit (Zymo Research) according to the manufacturer’s protocol, including a DNAse treatment. Extracted RNA was quantified by spectrophotometry (NanoDrop 1000, Fisher Scientific, Hampton, NH, USA) and quality checked by gel electrophoresis. Total RNA (1.0 μg) was reverse-transcribed to cDNA using SuperScript III Reverse Transcriptase (Life Technologies) in 20 μl reactions and primed with Oligo(dT)_20_ as per manufacturer’s protocol. Negative controls were performed in parallel by omitting RNA or enzyme. Obtained cDNA was diluted 1:10 before use and stored at -20°C. In order to search for candidate target genes, a microarray data set from developing Atlantic salmon [17] was screened for differentially expressed genes related to gut function. Few gut-related gene transcripts were found, probably because whole fish were used for RNA extraction. Furthermore, the vast number of differentially expressed genes appeared during embryonic stages, whereas only few genes were found to be differentially expressed after hatch. Therefore, we based our target gene list on a number of “classical” digestive enzyme and peptide hormone transcripts that have been widely used in studies of digestive tract development in marine fish larvae (*pep*, *trp-ia*, *bal*, *amy*, *ghrl*, *cck-l*, *pyy*). Additionally, we included several immune and digestion-related transcript markers with reported responses [18–23] during SBM-induced intestinal inflammation (*pept*, *sglt1*, *npc1l1*, *abcg5*, *apq8ab*, *il-17a*, *tcrγ*). The qPCR primers (Table 2) were obtained from the literature or designed using Primer3web (version 4.0.0, http://primer3.ut.ee/). PCR reaction efficiency for each gene assay was determined using 2-fold serial dilutions of pooled cDNA originating from randomly selected samples used in the experiment (starting dilution 1:5). The qPCR was run in the LightCycler 480 system (Roche Diagnostics) under the following conditions: Pre-incubation 95°C (5 min); amplification (40 cycles) 95°C (10 s); 60°C (10 s); 72°C (15 s); melt curve 95°C (5 s); 65°C (1 min); ramp to 97°C (0.11°/s, 5 acquisitions/degree). Each 10 μl DNA amplification reaction contained 2 μl PCR-grade water, 5 μl Lightcycler 480 SYBR Green I Master (Roche Diagnostics), 2 μl 1:10 diluted cDNA template and 0.5 μl (final concentration 500 nM) of each forward and reverse primer. Each sample was assayed in duplicate, including a no-template control and an inter-run plate calibrator. Quantification cycle (Cq) values were calculated using the second derivative method (Roche Diagnostics). Melt curve analysis and agarose gel inspection of products confirmed amplicons were only a single product. Eight potential reference genes were evaluated for use as a normalization factor (see [24] for details). Target gene expression was normalized to the geometric mean of *hprt1*, *actb* and *rnapolII*. Quantification of the target genes was performed using the ΔΔCq method [25].

**Table 2:** Primer pair sequences, amplicon size (AS; in basepairs [bp]), PCR efficiency (Eff.) and Genbank accession number (Acc. No.) for the genes used for quantitative real-time PCR.

Gene name	Forward (5’-3’)	AS	Eff.	Acc. No	Reference
	Reverse (5’-3’)	(bp)			
Trypsinogen 1a (*trp-ia*)	TACAAGTCCCGTGTGGAGGT	185	2.0	NM_001123711	16
	ACAGGCTGCACGTAGGTGTT				
Pepsinogen (*pep*)	GCCCTGTCCGAGTGTAATGT	164	2.0	NM_001160475	This study
	TCAGCATCGTTGGTCATAGC				
Amylase (*amy*)	AGGTGGCCGACTACATGAAC	184	2.0	NM_001123602	This study
	CCACCCATGTCGATAACCTC				
Bile salt-activated lipase (*bal*)	GCCAGTCATGGTTTGGATCT	121	2.0	L23929	This study
	CACGATAACTTTGCCCCTGT				
Peptide transporter (*pept*)	GGCTTTCTGCTCTGTGAAGG	89	1.9	EB174326	18
	TAGGGGGACACAACAAGACC				
Sodium/glucose cotransporter 1 (*sglt1*)	TCGTGGGATCTTTCATCCTCA	78	2.0	NM_001171787	18
	CCATGTAGCCCGTCTGGAAG				
Aquaporin 8ab (*aqp8ab*)	GTTGGCATAGTTCTCCTTTGATG	148	2.0	KC626879	19
	TTTCAACCCTCCCTTCACC				
ATP-binding cassette G5 (*abcg5*)	AGACTGCCTCGTCCAACACT	157	1.9	CU073172	20
	CCATTTTCGTGAACGTGTACC				
Cluster of differentiation 36 (*cd36*)	CAAGTCAGCGACAAACCAGA	91	1.9	NM_001124511	21
	AGGAGACATGGCGATGTAGG				
Nieman Pick C1-like1 (*npc1l1*)	CCAAAGACCTGATCCTGGAA	108	1.9	CB505644	22
	CGAAGCACACATCCTTCAGA				
Ghrelin (*ghrl*)*	CCCTTCACCAGGAAGACAAA	93	2.0	NM_001142709	This study
	CGGCACCATACTCCTGAAAC				
Cholecystokinin L (*cck-l*)^#^	CCTGAGCAGCAGAGCCAGCG	151	2.0	NM_001139521	This study
	GGTCCGTATGTTTCTATGAGGA				
Peptide yy (*pyy*)	AAGCCAAGCAAGAGAATCCA	112	2.0	NM_001139523	This study
	GACGGTCCAGGGTTTCAGTA				
Interleukin 17A (*il17a*)	AGGGGACAAAGGAGAGGTGT	114	2.0	GW574233	This study
	GGTGACAGAGAGCGTGTGTG				
T cell receptor gamma (*tcrγ*)	AGGCAGCAATCAACGAAAACC	116	1.9	EU221166	23
	GCTTGACCAAGTCTGGAAACA				

* Two Atlantic salmon ghrl isoforms have been reported [39]. The primer set used in this study has a perfect match to both isoforms, and will therefore not discriminate between them.

^#^ The *cck-l* isoform was chosen based on higher expression in the intestine [39].

### Digestive enzyme activities and bile salt concentrations

From all 18 time points, one pooled sample of 15 whole fish from each of the three replicate tanks per diet (n = 3 per diet) was used for the analyses. Trypsin activity was analyzed as described by Kakade et al. [26] and modified for Atlantic salmon as described in Krogdahl et al. [13] with N_α_-benzoyl-L-arginine 4-nitroanilide hydrochloride (L-BAPNA) as substrate. Trypsin activity is given in ΔOD per mg protein. Activity of leucine aminopeptidase (LAP) was determined using the substrate L-leucine-β-naphthylamide as described by Krogdahl et al. [13]. Maltase activity was determined according to methods described for disaccharidases by Dahlqvist [27] using maltose as the substrate. The activities of maltase and LAP are expressed as total activity per kg fish (Maltase: μmol/h/kg; LAP: mmol/h/kg). Bile salt levels were determined colorimetrically by using the Enzabile kit (Nycomed Pharma AS Diagnotics, Oslo, Norway). The analysis was performed on an Advia 1800 (Siemens Healthcare Diagnostics Erlangen, Germany) at the Central Laboratory of NMBU’s School of Veterinary Medicine, Oslo, Norway.

### Statistics

Statistical analyses were carried out using JMP Statistical software (SAS Institute, Cary, NC, USA). Unless otherwise indicated, pooled tank samples or tank means were used for the statistical analyses (n = 3). All data were tested for normality and homogeneity of variance, and where necessary data were transformed to improve the normality of distribution. Diet (FM/SBM) and time (dph) were evaluated as class variables in a two-way ANOVA with interaction. In addition, effect of time was evaluated for each diet separately by one-way ANOVA followed by Tukey’s multiple comparison test and results are presented as Supporting Information (S1, S2, S3 Tables). The level of significance for all analyses was set at *p*<0.05.

## Results

### Diets

Analyzed proximate compositions of the experimental diets were similar to expected compositions (Table 1). As a result of the higher fibre content of SBM compared to fishmeal, the SBM-containing diets were somewhat lower in protein and lipid, and therefore less energy dense than the FM diet. The protein-to-energy ratios of the diets were, however, similar and close to the predicted 25 g/MJ.

### Development

#### Survival and growth

**Fig 1:**
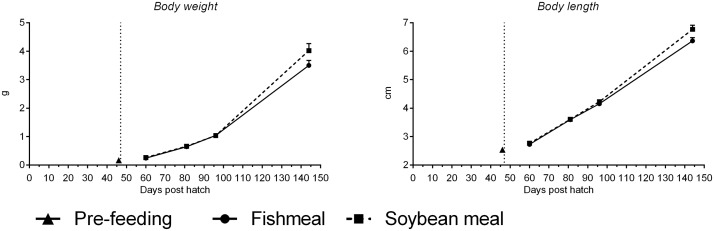
Mean body weight (g; left figure) and length (cm; right figure) of juvenile Atlantic salmon as a function of time (days post hatch) just before start-feeding at 46 days post hatch (indicated by vertical dotted line) and the period following start-feeding with the experimental diets (fishmeal or soybean meal diets). Values are means of n = 30–51 individual fish with standard error represented by vertical bars. See also S1 Table for mean values and results of post-hoc one-way ANOVA with time as the main variable.

Before start-feeding was initiated at 46 dph, mean fish body weight was 0.17 ± 0.002 g and mean body length was 2.5 ± 0.01 cm (Fig 1, Table 3). Body weight and length of FM-fed fish increased to 3.5 ± 0.17 g and 6.4 ± 0.11 cm at 144 dph. Body weight and length of SBM-fed fish increased to 4.0 ± 0.25 g and 6.7 ± 0.15 cm at 144 dph. Accurate assessment of mortality could only be detected from start-feeding (46 dph) and from this time point, very low mortality was observed during the feeding trial with a mean of 1.7% (pooled standard error [SE] 0.10). A significant effect of time was observed (*p*<0.0001) with highest mortalities observed following the initiation of start-feeding up to 96 dph and lowest mortalities during the period 97–144 dph. No effect of diet was observed (*p* = 0.431).

**Table 3:** Results (*p*-values) of the two-way ANOVA testing for the effect of diet (FM/SBM) and time (days post hatch), as well as their interaction (Diet*Time) on the various parameters.

Parameters	Diet	Time	Diet*Time
Mortality (number of fish)	0.431	<0.001*	0.301
Body weight (g)	0.913	<0.001*	0.003*
Body length (cm)	0.763	<0.001*	0.061
*Gene expression*			
Amylase	0.765	<0.001*	0.759
Aquaporin 8ab	0.816	<0.001*	<0.001*
ATP-binding cassette g5	0.711	<0.001*	0.020*
Bile salt-activated lipase	0.824	<0.001*	0.461
Cholecystokinin L	0.471	<0.001*	0.066
CD36	0.932	0.388	0.408
Ghrelin	0.177	0.014*	0.221
Interleukin 17a	0.690	<0.001*	0.111
Niemann-pick 1 like 1	0.730	<0.001*	0.108
Pepsin	0.606	<0.001*	0.079
Peptide transporter	0.999	<0.001*	0.083
Peptide YY	0.759	0.005*	0.268
Sodium-glucose transporter 1	0.922	<0.001*	0.070
T cell receptor gamma	0.580	<0.001*	0.059
Trypsinogen 1a	0.764	<0.001*	0.171
*Enzyme activities and bile concentration*			
Leucine aminopeptidase	0.188	<0.001*	0.312
Maltase	0.855	<0.001*	0.174
Trypsin	0.910	<0.001*	0.932
Bile salt concentration	0.950	<0.001*	0.017*

*Indicates significant effect (p < 0.05).

#### Histology

**Fig 2:**
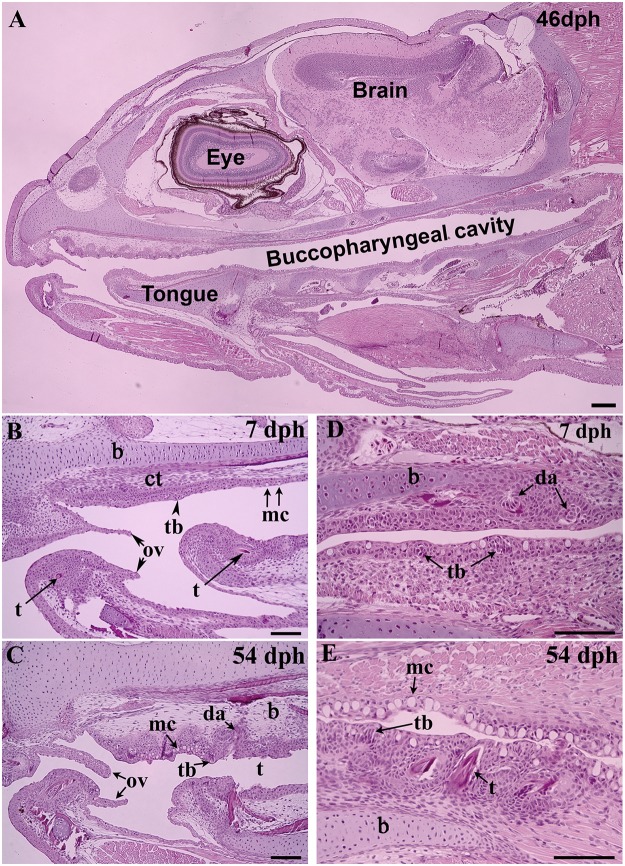
Representative sagittal sections of the buccopharyngeal cavity of Atlantic salmon juveniles at various time points (days post hatch [dph]). **(A)** General overview of alevin head at 46 dph; **(B-C)** Development of anterior region of oral cavity: two visible oral valves, squamous epithelium scattered with mucus cells and taste buds, presence of mandibular teeth and lingual teeth at 7 dph (B); increase in number of mucus cells, taste buds and teeth at 54 dph (C); **(D-E)** Development of pharynx: squamous epithelium scattered with mucus cells and taste buds, and presence of pharyngeal teeth indicating by the dental alveoli at 7 dph (D); large increased number of mucus cells, taste buds and pharyngeal teeth at 54 dph. Abbreviations: b, bone; ct, connective tissue; da, dental alveolus; mc, mucus cell; ov, oral valve; t, tooth; tb, taste bud. Scale bar: (A) = 400 μm; (B-E) = 50 μm.

Buccopharyngeal cavity (Fig 2): At 7 dph, the mouth had opened, and an anterior oral valve and tongue were clearly visible (Fig 2B and 2D). The buccopharyngeal cavity was lined by a simple squamous epithelium with scattered mucus cells, mostly found in the pharyngeal epithelium. Taste buds had appeared and were scattered within the epithelium. Underneath the epithelium was a thin layer of connective tissue. Dental alveoli were observed in the bones of the upper and lower jaw, and pharyngeal and lingual teeth also developed in the connective tissue and protruded into the buccopharyngeal lumen. At later time points (27–144 dph), no remarkable histological modifications were observed within the buccopharygeal cavity with the exception of an increase in number and size of mucus cells, taste buds, and teeth (Fig 2A, 2C and 2E).

**Fig 3:**
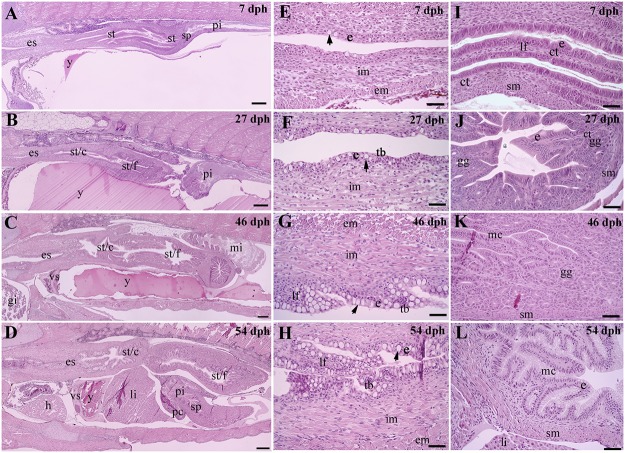
Representative sagittal sections of esophagus and stomach of Atlantic salmon juveniles at various time points (days post hatch [dph]). **(A-D)** General overviews of esophagus and stomach at 7, 27, 46, and 54 dph; **(E-H)** Development of the esophagus: simple epithelium with stratified squamous cells and mucus cells (arrows) at 7 and 27 dph (E and F), presence of taste buds within the epithelium at 27, 46 and 54 dph (F-H), presence of longitudinal mucosal folds at 46 and 54 dph (G and H), and increasing numbers of mucus cells (arrows) and thickness of internal longitudinal and external circular striated muscle over the time; **(I-L)** Development of the stomach: longitudinal folded mucosal consisting of simple columnar epithelium and connective tissue at 7 dph (I), presence of incipient gastric gland devoid of pepsinogen granules (J), presence of abundant pepsinogen granules in oxynticopeptic cells of gastric glands at 46 dph (K), and cardic glands lined by mucus cells at 54 dph (L). Abbreviations: ct, connective tissue; e, epithelium; em, external circular striated muscle; es, esophagus; gg, gastric gland made up of pepsinogen granule-containing oxynticopeptic cells; h, heart; im, internal longitudinal striated muscle; lf, longitudinal fold; li, liver; mc, mucus cell; mi, mid intestine; pc, pyloric caeca; pi, proximal intestine; sm, smooth muscle; sp, sphincter; st, stomach, st/c, stomach/cardia, st/f, stomach/fundus; tb, taste buds; vs, vitelline syncytium with vitelline veins; y, yolk. Scale bar: (A-L) = 50 μm.

Esophagus (Fig 3): At 7 dph, the esophagus had differentiated as a short, rudimentary duct that connected the posterior region of the pharynx and incipient stomach (Fig 3A and 3E). The esophagus was lined by a simple epithelium composed of stratified squamous cells. Scattered mucus cells were located within the epithelium. In the region towards the stomach, small longitudinal folds had formed. Two layers of internal longitudinal and external circular striated muscle surrounded the epithelium of the esophagus. At 27 dph, taste buds were detected within the epithelium (Fig 3B and 3F). At 46 dph, incipient longitudinally folded epithelium in the anterior region of the esophagus had developed. Mucus cells increased notably in abundance (Fig 3C and 3G). From 54–144 dph, longitudinal folds had grown in size (Fig 3D and 3H). The number of taste buds and thickness of the muscle layers had increased.

Stomach (Fig 3): At 7 dph, an early, straight stomach devoid of gastric glands was recognized as a bulge with longitudinal mucosal folds composed of columnar epithelium and connective tissue (Fig 3A and 3I). The primordial pyloric sphincter was developed and divided the stomach from the intestine. The submucosa consisted of a layer of connective tissue. The outer layer of the stomach was composed of external circular bundles of muscle tissue, which were transformed from striated skeletal muscle at the anterior end of the stomach to smooth muscle throughout the rest of the region. At 27 dph, the stomach had a discernable cardia, where the epithelium consisted of columnar cells, and fundus, where gastric glands had started to appear (Fig 3B and 3J). The gastric glands were made up of oxynticopeptic cells and surrounded by a thin layer of connective tissue. Pepsinogen granules in the oxynticopeptic cells were absent. At 46 dph, the stomach was bent in a “U” shape (Fig 3C). Mucus cells were present in the epithelium along the full length of the stomach (Fig 3K). The fundic area of the stomach was well developed with abundant tubular gastric glands. Eosinophilic pepsinogen granules were abundant in the oxynticopeptic cells (Fig 3C and 3K). From 54–144 dph, no major histological modifications were observed with the exception of stomach size, mucosal complexity, and thickness of the muscularis (Fig 3D and 3L).

**Fig 4:**
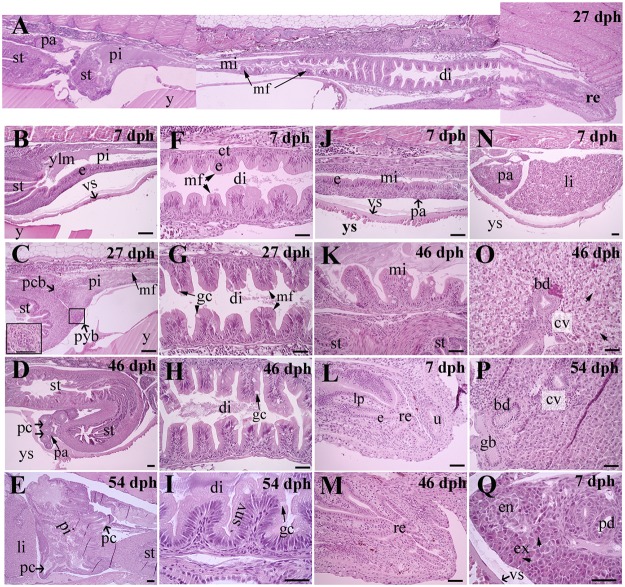
Representative sagittal sections of intestine, liver and pancreas of Atlantic salmon juveniles at various time points (days post hatch [dph]). **(A)** General overview of the intestine at 27 dph; **(B-E)** Development of the proximal intestine; absence of pyloric caeca at 7 dph (B), appearance of pyloric caeca buds at 27 dph (C), and more developed pyloric caeca at 46 and 54 dph (D and E); **(F-I)** Development of the distal intestine; presence of incipient mucosal folds without goblet cells at 7 dph (F), appearance of mucosal folds scattered with goblet cells (arrows) within epithelium at 27, 46 and 54 dph (G-I), and presence of low level of supranuclear vacuoles at 54 dph (I); **(J-K)** Development of the mid intestine; simple epithelium without goblet cells at 7 dph (J), and following appearance of mucosal folds scattered with goblet cells at 46 dph (K); **(L-M)** Development of the rectum (re); epithelium consisting of cuboidal cells devoid of mucus cells at 7 (L) and 46 (M) dph; **(N-P)** Development of the liver (li) consisting of polygonal hepatocytes with a prominent basophilic nucleus and eosinophilic cytoplasm irregularly arranged along the sinusoids around a central vein; increasing accumulation of glycogen-containing vacuoles (arrows) in hepatocytes from 7 (N) to 46 (O) dph; low level of glycogen deposit in liver following start-feeding at 54 dph (P); **(Q)** Fully differentiated pancreatic tissue composed of exocrine cells with zymogen granules (arrows), endocrine cells grouped in Langerhans islets, and pancreatic ducts at 7 dph. Abbreviations: bd, bile duct; ct, connective tissue; cv, central vein; di, distal intestine; e, epithelium; en, endocrine pancreas (Langerhans islet); ex, exocrine pancreas; gb, gallbladder; gc, goblet cell; li, liver; lp, lamina propria containing connective tissue; mf, mucosal folds; mi, mid intestine; pa, pancreas; pc, pyloric caeca with simple, columnar epithelium and goblet cells; pd, pancreatic duct; pi, proximal intestine; pyb, pyloric caeca bud; re, rectum; snv, supranuclear vacuoles; st, stomach; u, urinary opening; vs, vitelline syncytium; y, yolk; ylm, yolk-like material; ys, yolk sac. Scale bar: (B-E) = 100 μm or (C, insert) = 50 μm; (F-Q) = 50 μm.

Intestine (Fig 4): The intestine was consistently the longest portion of the digestive tract (Fig 4A). At 7 dph, the intestine was a simple straight tube, distinguishable as proximal and distal regions by the presence of mucosal folds in the latter region (Fig 4F), and rectum (Fig 4L). The proximal and distal regions were both lined by a simple epithelium with stratified columnar cells, whereas goblet cells were absent (Fig 4B and 4F). The mucosa was surrounded by a thin layer of connective tissue and longitudinal smooth muscle cells. Homogenous, eosinophilic, yolk-like material was seen in the lumen (Fig 4B). The short rectum was lined by a folded cuboidal epithelium devoid of goblet cells (Fig 4L) and the anus was open. The lamina propria consisted of loose connective tissue (Fig 4L). At 27 dph, the intestine was still straight (Fig 4A). Pyloric caeca buds had appeared in the anterior region (Fig 4C). A simple, folded mucosa had formed along the entire intestine, with longer folds located in the distal intestine (Fig 4A, 4C and 4G). Goblet cells had appeared within the epithelium (Fig 4G). At 46 dph, the anterior regions could be distinguished into proximal intestine equipped with pyloric caeca (Fig 4D) and mid intestine (Fig 4K). The mucosal folds of the distal intestine appeared longer and equipped with more goblet cells (Fig 4H). From the end of the proximal intestine, the elongated intestine had started to coil dorsally and continued distally to form an intestinal loop. The mucosa of the pyloric caeca consisted of a simple epithelium with stratified columnar cells along with scattered goblet cells (Fig 4D). Thin layers of connective tissue and smooth muscle cells were observed below the mucosa. Pancreatic tissue was observed in the mesenterium adjacent to the pyloric caeca (Fig 4D). From 54–144 dph, no major changes in the histomorphological structure of the intestine were observed with the exception of an increase in size of the intestine and increased height of mucosal folds (Fig 4E, 4I and 4M). Small supranuclear vacuoles were observed in the distal intestinal enterocytes in three of nine fish (Fig 4I).

Liver and pancreas (Fig 4): At 7 dph, signs of functional liver and pancreatic tissue cells were visible (Fig 4N and 4Q). In the liver, polygonal hepatocytes with a prominent basophilic nucleus and eosinophilic cytoplasm were irregularly arranged along the sinusoids around a central vein. Small glycogen vacuoles were present in the cytoplasm of the hepatocytes and vacuolization increased continuously until 46 dph (Fig 4N and 4O). With the increased vacuolization, the nucleus gradually shifted toward the periphery of the hepatocytes. At 54 dph, however, the presence of vacuoles was low and similar to the level at 7 dph (Fig 4P).

The pancreas was diffusely arranged within the mesenteric tissue, initially in the entire abdominal cavity. As the intestinal tract lengthened and started to fold, most was found located adjacent to the anterior intestinal regions (data not shown). From 7 dph, the exocrine pancreas was organized in acini, which consisted of a single layer of pyramidal cells containing clearly visible eosinophilic zymogen granules (Fig 4Q). Pancreatic acini were grouped around pancreatic ducts, which were well developed and lined with columnar epithelial cells. The cells of the endocrine pancreas, grouped in a number of Langerhans islets, were visible and grouped among the exocrine pancreatic cells (Fig 4Q).

Yolk sac (Fig 4): At 7 and 27 dph, the yolk sac was attached ventrally to the digestive tract and occupied most of the abdominal cavity. The yolk consisted of a large amount of homogeneous eosinophilic material and a number of oil droplets. The entire surface of the yolk was enveloped by a thin syncytial tissue, the vitelline syncytium (Fig 4J), through which vitelline veins were distributed. At 7 dph, yolk-like homogenous eosinophilic material was also observed within the intestinal lumen (Fig 4B). The yolk sac progressively decreased in size over time. The remainder of the yolk sac, still connected with larger vitelline veins, was gradually restricted to the anterior region of the abdominal cavity. The vitelline syncytium was then thickened and contained a number of amoeboid nuclei (data not shown).

#### Gene expression

**Fig 5:**
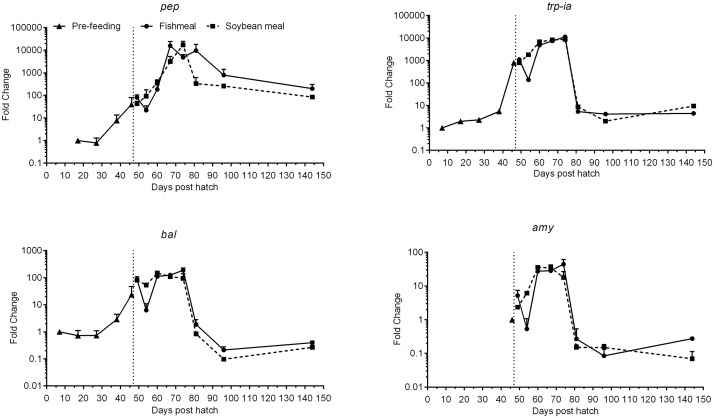
Development of the expression of gastric (*pep*) and pancreatic (*trp-ia*, *bal* and *amy*) digestive enzyme genes of juvenile Atlantic salmon as a function of time (days post hatch), differentiated by the pre-feeding period and the period following start-feeding (indicated by the vertical dotted line at 46 days post hatch) with the experimental diets (fishmeal or soybean meal diets). Values are mean fold change (log_10_) as compared to the respective time point of first detection; n = 3 pooled samples of 3 individual fish per tank (three tanks per diet) with standard error represented by vertical bars. Abbreviations: *pep*, pepsinogen; *trp-ia*, trypsinogen 1a; *bal*, bile salt-activated lipase; *amy*, alpha-amylase. See also S2 Table for mean values and results of post-hoc one-way ANOVA with time as the main variable.

Pepsinogen (*pep*), trypsinogen 1a (*trp-ia*), bile salt activated lipase (*bal*) and amylase (*amy*) expression showed an overall similar trend in their development throughout the experiment (Fig 5, Table 3). While *trp-ia* and *bal* were detected at low levels from 7–27 dph, *pep* expression was first detectable at 17 dph and *amy* at 46 dph. *Trp-ia*, *pep* and *bal* expression increased markedly from 38 to 46 dph, i.e. just before the onset of exogenous feeding, and remained high during the first 32 days of exogenous feeding, peaking at 67–74 dph. *Amy* expression increased on the first day of exogenous feeding and also remained at elevated levels for the first 32 days of feeding, also peaking at 67–74 dph. At 81 dph, mRNA expression of the digestive enzymes decreased to levels similar to the first detectable levels observed (at 7–46 dph), except for *pep*, which remained >100-fold higher than 17 dph levels. The magnitude of change was highest for *pep*, with >20,000-fold induction at time points 67–81 dph compared to 7 dph. *Trp-ia* also showed levels differing by more than three orders of magnitude.

**Fig 6:**
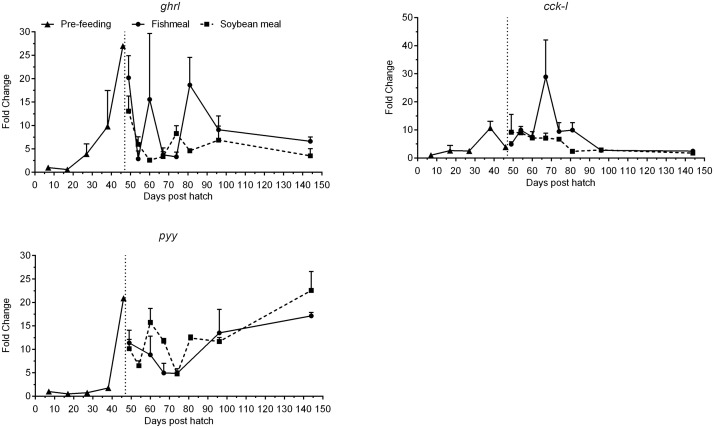
Development of the expression of signaling peptide genes of juvenile Atlantic salmon as a function of time (days post hatch), differentiated by the pre-feeding period and the period following start-feeding (indicated by the vertical dotted line at 46 days post hatch) with the experimental diets (fishmeal or soybean meal diets). Values are mean fold change as compared to the respective time point of first detection; n = 3 pooled samples of 3 individual fish per tank (three tanks per diet) with standard error represented by vertical bars. Abbreviations: *ghrl*, ghrelin; *cck-I*, cholecystokinin L; *pyy*, peptide yy. See also S2 Table for mean values and results of post-hoc one-way ANOVA with time as the main variable.

Ghrelin (*ghrl*) mRNA expression levels were very low until 17 dph (Fig 6, Table 3). From 27 dph levels increased, peaked at 46 dph and remained elevated by >6-fold compared to 7 dph throughout the experimental period. Cholecystokinin-L (*cck-l*) was expressed at low levels until 27 dph and subsequently increased until it peaked on 70 dph and then decreased progressively until the end of the experiment to levels ca. 2-fold greater than 7 dph (Fig 6, Table 3). Expression levels of peptide yy (*pyy*) were very low until 38 dph and showed an increasing pattern thereafter, with expression levels at 144 dph >10-fold higher than at 7 dph (Fig 6, Table 3). Both *pyy* and *ghrl* had a peak in expression at 46 dph, just before feeding was initiated.

**Fig 7:**
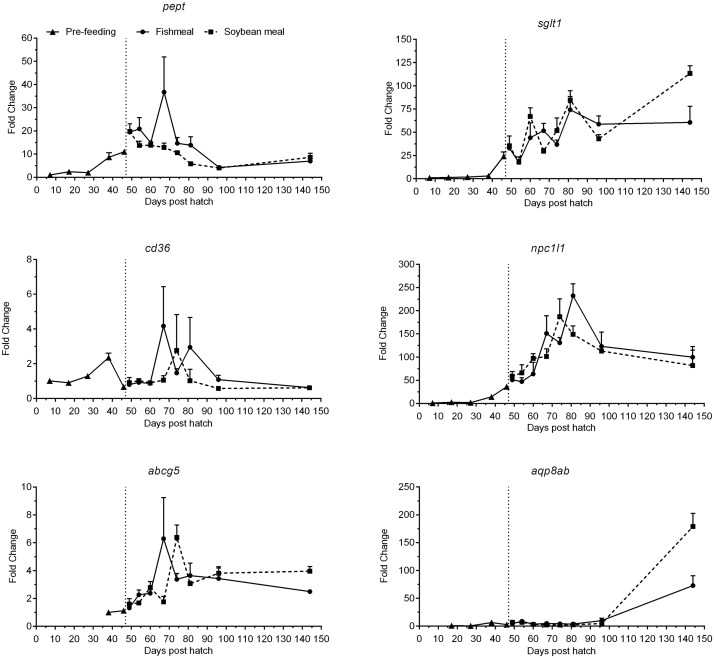
Development of the expression of transporter genes of juvenile Atlantic salmon as a function of time (days post hatch), differentiated by the pre-feeding period and the period following start-feeding (indicated by the vertical dotted line at 46 days post hatch) with the experimental diets (fishmeal or soybean meal diets). Values are mean fold change as compared to the respective time point of first detection; n = 3 pooled samples of 3 individual fish per tank (three tanks per diet) with standard error represented by vertical bars. Abbreviations: *pept*, peptide transporter; *sglt1*, sodium/glucose cotransporter 1; *cd36*, cluster of differentiation 36; *npc1l1*, Nieman Pick C1-like 1; *abcg5*, ATP-binding cassette G5; *aqp8ab*, aquaporin 8ab. See also S2 Table for mean values and results of post-hoc one-way ANOVA with time as the main variable.

Peptide transporter (*pept*) was expressed at low levels until 27 dph (Fig 7, Table 3). It increased steadily until 3 days post start-feeding (49 dph) and subsequently decreased steadily and leveled off after 96 dph. At 144 dph, expression levels were >7-fold higher than at 7 dph. Expression levels of sodium glucose cotransporter (*sglt1*) were very low until 38 dph (Fig 7, Table 3). Thereafter, a continuous and strong increase throughout the experimental period was seen, with expression at 144 dph >60-fold higher than at 7 dph. Expression levels of the fatty acid transporter Cluster of differentiation 36 (*cd36*) showed a peak at 38 dph and decreased to initial levels during the first days of exogenous feeding until 60 dph (Fig 7, Table 3). Thereafter, the expression varied strongly but at 144 dph had receded to levels comparable to 7 dph. Expression levels of the cholesterol transporter niemann-pick C1-like 1 (*npc1l1*) were very low until 27 dph but increased markedly thereafter, peaking at 74–81 dph and apparently leveling off at 96–144 dph. Levels at 144 dph were ca. 90-fold higher compared to 7 dph (Fig 7, Table 3). A similar pattern was observed for the sterol efflux transporter ATP-binding cassette g5 (*abcg5*), but the magnitude of change was smaller and expression levels were below detection limits before 38 dph (Fig 7, Table 3). Compared to 38 dph, expression levels were 2–4 fold higher at 144 dph. Aquaporin 8ab (*aqp8ab*) expression was first detectable at 17 dph at very low levels, and remained low until 96 dph, with a subsequent strong increase observed at the end of the experimental period to levels >70-fold higher than at 17 dph (Fig 7, Table 3).

**Fig 8:**
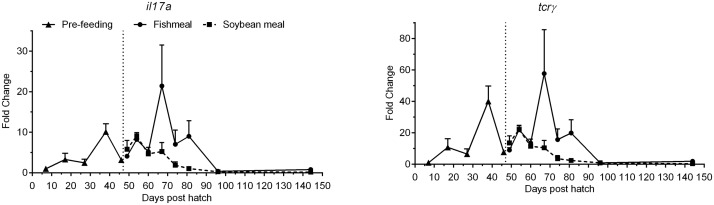
Development of the expression of T-cell marker genes of juvenile Atlantic salmon as a function of time (days post hatch), differentiated by the pre-feeding period and the period following start-feeding (indicated by the vertical dotted line at 46 days post hatch) with the experimental diets (fishmeal or soybean meal diets). Values are mean fold change as compared to the respective time point of first detection; n = 3 pooled samples of 3 individual fish per tank (three tanks per diet) with standard error represented by vertical bars. Abbreviations: *il17a*, interleukin 17A; *tcrγ*, T cell receptor gamma. See also S2 Table for mean values and results of post-hoc one-way ANOVA with time as the main variable.

The expression profiles of T cell receptor gamma (*tcrγ*) and interleukin 17a (*il17a*) showed parallel expression patterns (Fig 8, Table 3). Expression was detectable from 7 dph and showed a variable but overall increasing pattern, peaking at 67 dph before decreasing. Low expression levels comparable to 7 dph were observed at 96 and 144 dph.

#### Enzyme activities and bile salt concentration

**Fig 9:**
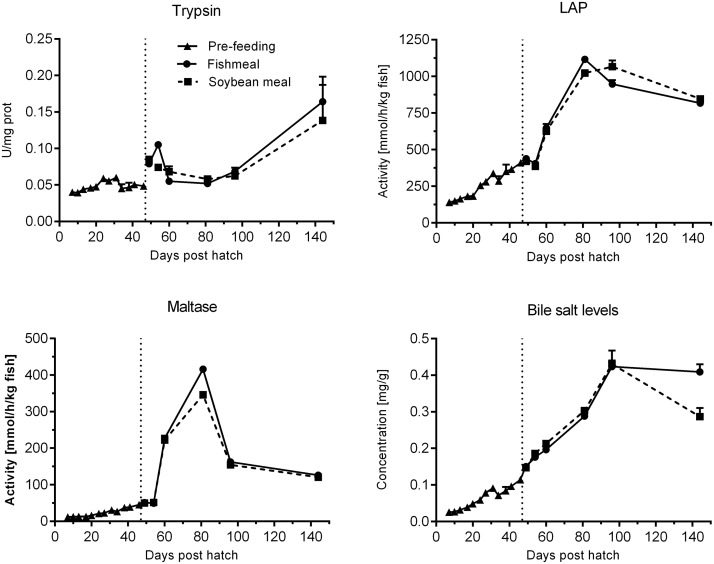
Development of digestive enzyme activities for pancreatic trypsin and brush border membrane enzymes leucine aminopeptidase (LAP) and maltase as well as bile salt levels of juvenile Atlantic salmon as a function of time (days post hatch), differentiated by the pre-feeding period and the period following start-feeding (indicated by the vertical dotted line at 46 days post hatch) with the experimental diets (fishmeal or soybean meal diets). Values are means (n = 3 pooled samples of 15 individual fish per tank; three tanks per diet) with standard error represented by vertical bars. See also S3 Table for mean values and results of post-hoc one-way ANOVA with time as the main variable.

Trypsin activity remained low and relatively stable before start-feeding (Fig 9, Table 3). The activity increased 3 days post start-feeding (49 dph) and displayed a relatively stable pattern until 96 dph. A marked increase was observed from 96 to 144 dph to levels 4-fold higher than at 7 dph. Maltase activities were low but steadily increased from 7 until 54 dph (Fig 9, Table 3). A stronger increase in activity was observed after 54 dph, peaking at 81 dph with a subsequent decrease toward 144 dph. At the end of the experimental period, maltase levels were >10-fold higher than at 7 dph. Leucine aminopeptidase displayed higher activity levels than maltase by 3–12 fold. Otherwise the pattern of development for the two brush border membrane enzymes was similar with an increasing trend during the experimental period (Fig 9, Table 3). Again, a stronger increase in activity was apparent from 8 days post start-feeding (54 dph), peaking at 81 dph, before apparently leveling off at 96 and 144 dph to levels 6-fold higher than 7 dph. Whole body bile salt levels increased steadily from 7 to 96 dph, but seemed to level off towards the end of the experiment to levels 11–16 fold higher than 7 dph (Fig 9, Table 3).

### Diet effect

Fish that received the 16.7% SBM diet did not significantly differ in growth or survival compared to FM-fed fish (Table 3). Total cumulative mortality tended (*p* = 0.0523) to be higher in the FM (mean 1.9%) than in the SBM (1.5%; pooled SE 0.10) fed fish. No differences in histomorphological development were observed and no signs of inflammatory responses were visible in the intestine or other organs of any fish fed either diet. The investigated genes did not show significant differences between FM and SBM in their respective mRNA expression ratios. Nor did enzyme activities differ significantly between FM and SBM-fed fish. Furthermore, no trend was evident in the data that may indicate adverse developmental, functional or immunological SBM effects on mRNA expression or enzyme activities.

## Discussion

### Liver and pancreas

The liver and pancreas, as well as pancreatic and bile ducts, appeared to develop earlier than the other digestive organs, which is in line with descriptions in several other teleost species [28–31]. In Atlantic salmon, the pancreas is a diffuse organ, arranged in the mesenteric tissue surrounding the pyloric caeca. The pancreas is responsible for the synthesis of precursors of all major digestive enzymes that are necessary for luminal digestion, such as proteases (e.g. trypsinogen), glucosidases (e.g. α-amylase) and lipases (e.g. bile salt-dependent lipase). Expression levels of the pancreatic enzyme genes *trp-ia* and *bal* were up-regulated before exogenous feeding, which indicate concomitant development of zymogen granules in the exocrine pancreas in preparation for enzyme production. Alpha-amylase was an exception, as transcript levels were below detection level until shortly before start-feeding at 46 dph. In marine fish species, different patterns of *amy* expression during the larval stage have been observed, and the variations have been attributed to differences in rearing conditions and feed type [32]. Natural diet preferences may attribute to species differences in the importance of amylase for digestion in general. For piscivorous salmon, low or undetectable amylase activity has been reported in more mature salmon [33–35], which is apparently due to a seven amino acid deletion close to the active site, possibly impairing substrate binding, as well as unique characteristics in the signaling peptide that may negatively affect synthesis of the enzyme [34]. An inherently low need for amylase in Atlantic salmon may at least partially explain the low expression of *amy* in juveniles in the current study.

The gastrointestinal peptide hormone cholecystokinin (cck) induces the release of zymogen granules from the exocrine pancreas and bile from the gall bladder into the proximal intestine. Cholecystokinin secretion, in turn, is triggered by the presence of proteins, lipids and carbohydrates in the intestine [36]. Peptide YY (pyy) acts as an antagonistic peptide to cck by suppressing pancreatic secretion [37]. Thus, the release of zymogen granules is a tightly controlled process which may be reflected in our observation of fluctuating expression levels of *cck-l* and *pyy*, especially after the onset of exogenous feeding. Interestingly, *cck-l* and *pyy* seemed to display opposite expression profiles at later time points, which is in line with the antagonistic expression patterns observed after fasting in yellowtail, *Seriola quinqueradiata* [38]. In addition to their role in the regulation of digestive functions, cck and pyy play important roles in the control of feed intake [39]. There are no previous reports on ontogenetic expression patterns of *cck-l* and *pyy* in salmonids, but available results from marine fish species indicate that at least cck production in the gut is mainly genetically pre-programmed in early larval stages, but is influenced by luminal dietary factors after the yolk has been depleted [40].

Bile salt levels increased steadily from 7 dph, demonstrating significant hepatic bile production well before the onset of exogenous feeding. Early functional development of the liver was also indicated by increased accumulation of glycogen vacuoles within the hepatocytes. The liver is the main site for gluconeogenesis and plays an important part in energy metabolism. Vacuole accumulation continued during the yolk sac stage, which is in line with a previous study in chum salmon [28]. The accumulation of glycogen in liver before start-feeding indicated that alevins converted and stored nutrients from the yolk sac. Moreover, the appearance of the Langerhans islets at an early stage has been suggested to contribute to hepatic glycogen biosynthesis in alevins [28].

### Stomach and pyloric caeca

The stomach and the pyloric caeca were the last features to develop morphologically, which is in line with studies in other teleosts [32,41–49]. Increasing expression of pepsinogen and the gastric signaling peptide ghrelin coincided with the onset of gastric gland formation at 27 dph. Ghrelin has multiple roles in mammals and fish, such as induction of growth hormone release, stimulation of appetite and gastric acid secretion [50–51]. Atlantic salmon ghrelin has been characterized and is mainly expressed in the stomach [39]. The qPCR assay used in the present work does not discriminate between the two reported *ghrl* isoforms, and will therefore not detect potential isoform-specific differential expression during salmon development. Isoform-specific regulation is, however, unlikely as increased expression of both identified *ghrl* isoforms before exogenous feeding has also previously been reported in salmon [52]. The major histological and molecular changes observed between 27 and 46 dph in our study, suggest that the stomach of Atlantic salmon alevins may be functional before the yolk sac is completely internalized at 46 dph. Similar observations have been reported in masu (cherry) salmon, *Oncorhynchus masou* B. [44], and chum salmon, *Oncorhynchus keta* W. [28], where a functional stomach was also observed before initiation of exogenous feeding. Thus, expression and functionality of pepsin and acid digestion appears to occur earlier in salmonids than in many marine species, such as white sturgeon, *Acipenser transmontanus* [53] and red porgy, *Pagrus pagrus* [54–55], in which these functions appeared to be initiated after start-feeding. In marine fish larvae, the appearance of a functional stomach is generally considered as the transition from the larval to the juvenile stage in fish, and has been used as a marker to initiate weaning onto formulated feed [32,56–57].

The formation of pyloric caeca drastically increases the mucosal surface area and thereby also the digestive capacity [43,58]. Since the major proportion of digestion and absorption of nutrients takes place in this region [35], their functional development may be considered an important marker for the development of the functional digestive system. Pyloric caeca were first observed at 27 dph as primitive buds originating from the anterior intestine. Coinciding with this, functional development of nutrient absorptive functions was indicated by increased transcript levels of the peptide transporter *pept*, Na+-glucose cotransporter *sglt1* and the cholesterol transporters *npc1l1* and *abcg5*. Activities of the brush border-associated enzymes LAP and maltase also steadily increased during the same time period, which indicated that the pyloric caeca and the rest of the intestine matured and attained luminal digestive capacity [59]. Taken together, these results suggest that the tissue’s ability to digest and absorb nutrients started to increase in parallel with the development of the pyloric caeca, and well before exogenous feeding was initiated. Interestingly, the presumed fatty acid transporter *cd36* was expressed at low levels and expression was apparently not influenced by the diet or development. In adult salmon, *cd36* is expressed at relatively high levels in the pyloric caeca, and expression is clearly altered depending on the degree of intestinal lipid accumulation [21]. This indicates a functional role of cd36 in lipid uptake in salmon, but other fatty acid transport proteins are also present, such as fatty acid transport proteins (fatps) and plasma membrane associated fatty acid binding protein (fabp_pm_). Fatty acid uptake seems to be dependent on both transporter-mediated and passive absorption [60]. According to knowledge from mammalian research, the relative importance of these two mechanisms is probably highly dependent on the microenvironment and tissue phenotype [61]. Therefore, the development of at least cd36 may not be needed or prioritized during early ontogeny of the salmon intestine.

### Mid and distal intestine

In addition to increased gene expression and activity levels of digestive enzymes discussed above, functional development of at least the distal intestine was also indicated by the formation of supranuclear vacuoles, as this is considered to be a sign of functioning protein pinocytosis in enterocytes [49,62–64]. The accumulation of supranuclear vacuoles in distal intestinal enterocytes has been observed in juvenile and adult Atlantic salmon [10,12–14], carp, *Cyprinus carpio* L. [64], *Tilapia* spp. [65], and zebrafish, *Danio rerio* [66,67]. The individual variability in the degree of vacuolization may be due to differences in feed intake since fasting has been shown to rapidly decrease vacuolization [12]. The ontogenetic expression profile of *aqp8ab* was different from other genes, as low levels were detected from 7–96 dph and gene expression was not affected by the transition from endogenous to exogenous feeding. Instead, *aqp8ab* was strongly up-regulated at the last time point (144 dph). Increased gene expression of this water channel may be an indication that the organism is beginning to prepare for the transfer to seawater (smoltification), and is in line with the previously reported induction during smoltification [68]. The particular importance of Aqp8 in regulating the intestinal water balance is further supported by the strongly affected *aqp8ab* mRNA levels in the distal intestine of salmon suffering from diet-induced inflammation with accompanying diarrhea [19]. Of note was also the observation of yolk-like material within the intestinal lumen of alevins 7 dph. Teleost fish apparently absorb their yolk through the yolk syncytium, and this is believed to occur without any involvement of the gut. In contrast, the majority of yolk is digested within the intestine of elasmobranchs [69]. The present study may indicate a mechanism more similar to elasmabranchs in Atlantic salmon, which deserves further attention.

### Effects of soybean meal

Contrary to post-smolt, seawater-adapted Atlantic salmon, high inclusion of SBM did not induce intestinal inflammation, nor did it negatively affect growth, survival, enzyme activities, bile salt concentration or gene expression of various key functional and T-cell markers in start-feeding fry. Nor were skeletal development or gene expression levels of other immune and cellular stress response parameters, recently reported from the same feeding trial [70], affected by SBM. Negative effects of SBM on intestinal health and function of seawater-adapted Atlantic salmon have been well documented over the past 20+ years [11–14,71–75]. However, studies on early developmental stages of Atlantic salmon are few. Results of a study investigating the effects of SBM on intestinal health in Atlantic salmon parr are in line with the present work, showing increased cell proliferation but no signs of the inflammatory response that is typically found in post-smolt salmon [10]. The cytokine *il17a* and the T-cell receptor *tcrγ* have been proposed as possible genetic markers for SBM-induced inflammation in salmon intestinal tissue [23]. In our study, expression levels of *il17a* and *tcrγ* were detectable from 7 dph and fluctuated before and after initiation of exogenous feeding, but no significant SBM effect was observed. In post-smolt Atlantic salmon, SBM and other plant protein sources also affect bile salt levels and transcription of genes related to their metabolism [19,20,73,74]. Furthermore, post-smolt Atlantic salmon affected by SBM-induced enteritis have significantly increased trypsin activity in distal intestinal content and tissue, whereas LAP and maltase activities are reduced [16,23,75], indicating general tissue dysfunction accompanying the inflammation. No SBM effects on these parameters in juveniles up to 144 dph were observed in the present study.

The reason for the lack of inflammatory response in the juvenile salmon is not clear. One possible explanation is that the under-developed adaptive immune system may not have been equipped to mount an inflammatory response. T-cells, apparently central in the SBM-induced inflammatory response in more developed salmon [23,71], as well as for mounting a protective immune response following vaccination, may not have been functional in these juvenile fish up to 144 dph and a body weight of about 4 g, despite detectable gene expression of *il17a* and *tcrγ*. This is supported by vaccination practices in salmon aquaculture, with a recommendation of minimum 15 g body weight at first vaccination to ensure a protective antibody response. Further investigations revealed that when the juveniles continued to receive the SBM-containing diet, they did start to develop distal intestinal inflammation as their functional immune system developed (Bakke, unpublished results).

### Conclusions

To our knowledge, this is the first detailed description of the ontogeny of the digestive system of Atlantic salmon for the first 5 months from hatch and following start-feeding. At 7 dph, the digestive system was morphologically recognizable with an early stomach, liver, pancreas, and anterior and posterior intestine, but no pyloric caeca were distinguishable. Functional development of the digestive organs occurred in the following sequence (from early to late): liver and pancreas > mouth and rectum > esophagus and intestine > stomach and pyloric caeca. Most mRNA levels of the investigated genes related to digestive functions were detectable at 7 dph and increased before exogenous feeding was initiated. Similarly, bile salt levels as well as pancreatic and brush border enzyme activities increased steadily from 7 dph. The work indicated that several aspects of the Atlantic salmon alevin digestive functions are prepared for digestion of external feed well before the yolk sac is internalized into the abdominal cavity. Furthermore, juvenile salmon appear to tolerate up to 16.7% extracted soybean meal in their diets for the first three months following start-feeding, which could indicate that SBM may be used during this period.

## Supporting Information

S1 Table Growth performance of juvenile Atlantic salmon from 46 to 144 days post hatch (dph). Values are means of n = 31–51 individual fish. P values for the one-way ANOVA are listed for each diet separately. Different superscript letters indicate time points that are significantly different within one diet according to Tukey's multiple comparison test.
(XLS)

S2 Table Gene expression of juvenile Atlantic salmon from 7 to 144 days post hatch (dph). Data are mean ΔΔCt values of n = 3 pooled samples of 3 individual fish per tank (three tanks per diet). P values for the one-way ANOVA are listed for each diet separately. Different superscript letters indicate time points that are significantly different within one diet according to Tukey's multiple comparison test.
(XLS)

S3 Table Enzyme activities and bile salt levels of juvenile Atlantic salmon from 7 to 144 days post hatch (dph). Values are means of n = 3 pooled samples of 15 individual fish per tank, three tanks per diet. P values for the one-way ANOVA are listed for each diet separately. Different superscript letters indicate time points that are significantly different within one diet according to Tukey's multiple comparison test.
(XLS)

## References

[1] Gunnes K. Survival and development of Atlantic salmon eggs and fry at three different temperatures. Aquaculture. 1979;16: 211–218.

[2] Heming TA. Effects of temperature on utilization of yolk by chinook salmon (*Oncorhynchus tshawytscha*) eggs and alevins. Can J Fish Aquat Sci. 1982;39: 184–190.

[3] Killeen J, McLay HA, Johnston IA. Development in *Salmo trutta* at different temperatures, with a quantitative scoring method for intraspecific comparisons. J Fish Biol. 1999;55: 382–404.

[4] Macqueen DJ, Robb DHF, Olsen T, Melstveit L, Paxton CGM, Johnston IA. Temperature until the 'eyed stage' of embryogenesis programmes the growth trajectory and muscle phenotype of adult Atlantic salmon. Biol Letters. 2008;4: 294–298. 10.1098/rsbl.2007.0620 PMC2610038 18348956

[5] Knoph MB. Acute toxicity of ammonia to Atlantic salmon (*Salmo salar*) parr. Comp Biochem Physiol C Comp Pharmacol. 1992;101: 275–282. 1354102 10.1016/0742-8413(92)90273-a

[6] Rombough PJ, Garside ET. Cadmium toxicity and accumulation in eggs and alevins of Atlantic salmon *Salmo salar* . Can J Zool. 1982;60: 2006–2014.

[7] Gorodilov YN. Description of the early ontogeny of the Atlantic salmon, *Salmo salar*, with a novel system of interval (state) identification. Environ Biol Fish. 1996;47: 109–127.

[8] Tacon AG, Hasan MR, Metian M. Demand and supply of feed ingredients for farmed fish and crustaceans: trends and prospects. FAO Fisheries and Aquaculture Technical Paper. 2011; 56487.

[9] Krogdahl Å, Penn MH, Thorsen J, Refstie S, Bakke AM. Important antinutrients in plant feedstuffs for aquaculture: an update on recent findings regarding responses in salmonids. Aquacult Res. 2010;41: 333–344.

[10] Sanden M, Berntssen MHG, Krogdahl Å, Hemre GI, Bakke-McKellep AM. An examination of the intestinal tract of Atlantic salmon, *Salmo salar* L., parr fed different varieties of soy and maize. J Fish Dis. 2005;28: 317–330. 15960655 10.1111/j.1365-2761.2005.00618.x

[11] van den Ingh TSGA, Krogdahl Å, Olli JJ, Hendriks HGCJ, Koninkx JGJF. Effects of soybean-containing diets on the proximal and distal intestine in Atlantic salmon (*Salmo salar*): a morphological study. Aquaculture. 1991;94: 297–305.

[12] Baeverfjord G, Krogdahl Å. Development and regression of soybean meal induced enteritis in Atlantic salmon, *Salmo salar* L, distal intestine: A comparison with the intestines of fasted fish. J Fish Dis. 1996;19: 375–387.

[13] Krogdahl Å, Bakke-McKellep AM, Baeverfjord G. Effects of graded levels of standard soybean meal on intestinal structure, mucosal enzyme activities, and pancreatic response in Atlantic salmon (*Salmo salar* L.). Aquacult Nutr. 2003;9: 361–371.

[14] Urán PA, Aydin R, Schrama JW, Verreth JAJ, Rombout JHWM. Soybean meal-induced uptake block in Atlantic salmon *Salmo salar* distal enterocytes. J Fish Biol. 2008;73: 2571–2579.

[15] NRC. Nutrient Requirements of Fish. Washington DC: National Academy Press Inc.; 1993.

[16] Lilleeng E, Frøystad MK, Østby GC, Valen EC, Krogdahl Å. Effects of diets containing soybean meal on trypsin mRNA expression and activity in Atlantic salmon (*Salmo salar* L). Comp Biochem Physiol A Mol Integr Physiol. 2007;147: 25–36. 17293147 10.1016/j.cbpa.2006.10.043

[17] Jantzen SG, Sanderson DS, von Schalburg KR, Yasuike M, Masass F, Koop BF. A 44K microarray dataset of the changing transcriptome in developing Atlantic salmon (*Salmo salar* L.). BMC Res Notes. 2011;4: 88 10.1186/1756-0500-4-88 21447175 PMC3073910

[18] Gu J, Krogdahl Å, Sissener NH, Kortner TM, Gelenscer E, Hemre G-I, et al Effects of oral *Bt*-maize (MON810) exposure on growth and health parameters in normal and sensitised Atlantic salmon, *Salmo salar* L. Br J Nutr. 2013;109: 1408–1423. 10.1017/S000711451200325X 23182224

[19] Kortner TM, Skugor S, Penn MH, Mydland LT, Djordjevic B, Hillestad M, et al Dietary soyasaponin supplementation to pea protein concentrate reveals nutrigenomic interactions underlying enteropathy in Atlantic salmon (*Salmo salar*). BMC Vet Res. 2012;8: 101 10.1186/1746-6148-8-101 22748053 PMC3424111

[20] Kortner TM, Gu J, Krogdahl Å, Bakke AM. Transcriptional regulation of cholesterol and bile acid metabolism after dietary soyabean meal treatment in Atlantic salmon (*Salmo salar* L.). Br J Nutr. 2013;109: 593–604. 10.1017/S0007114512002024 22647297

[21] Gu M, Kortner TM, Penn M, Hansen AK, Krogdahl Å. Effects of dietary plant meal and soya-saponin supplementation on intestinal and hepatic lipid droplet accumulation and lipoprotein and sterol metabolism in Atlantic salmon (*Salmo salar* L.). Br J Nutr. 2014;111: 432–444. 10.1017/S0007114513002717 24507758

[22] Kortner TM, Björkhem I, Krasnov A, Timmerhaus G, Krogdahl Å. Dietary cholesterol supplementation to a plant-based diet suppresses the complete pathway of cholesterol synthesis and induces bile acid production in Atlantic salmon (*Salmo salar* L.). Br J Nutr. 2014;111: 2089–2103. 10.1017/S0007114514000373 24635969

[23] Marjara IS, Chikwati EM, Valen EC, Krogdahl Å, Bakke AM. Transcriptional regulation of IL-17A and other inflammatory markers during the development of soybean meal-induced enteropathy in the distal intestine of Atlantic salmon (*Salmo salar* L.). Cytokine. 2012;60: 186–196. 10.1016/j.cyto.2012.05.027 22795954

[24] Kortner TM, Valen EC, Kortner H, Marjara IS, Krogdahl Å, Bakke AM. Candidate reference genes for quantitative real-time PCR (qPCR) assays during development of a diet-related enteropathy in Atlantic salmon (*Salmo salar* L.) and the potential pitfalls of uncritical use of normalization software tools. Aquaculture. 2011;318: 355–363.

[25] Livak KJ, Schmittgen TD. Analysis of relative gene expression data using real-time quantitative PCR and the 2(-Delta Delta C(T)) method. Methods. 2001;25: 402–408. 11846609 10.1006/meth.2001.1262

[26] Kakade ML, Hoffa DE, Liener IE. Contribution of trypsin inhibitors to the deleterious effects of unheated soybeans fed to rats. J Nutr. 1973;103: 1772–1778. 4796250 10.1093/jn/103.12.1772

[27] Dahlquist A. Assay of intestinal disaccharidases. Enzym Biol Clin. 1970;11: 52–66. 5414130

[28] Takahashi K, Hatta N, Sugawara Y. Organogenesis and functional revelation of alimentary tract and kidney of chum salmon. Tohoku J Agricult Res. 1978;29: 98–109.

[29] Bisbal GA, Bengtson DA. Development of the digestive tract in larval summer flounder. J Fish Biol. 1995;47: 277–291.

[30] Guyot E, Diaz JP, Connes R. Organogenesis of the liver in sea bream. J Fish Biol. 1995;47: 427–437.

[31] Pena R, Dumas S, Villalejo-Fuerte M, Ortiz-Galindo JL. Ontogenetic development of the digestive tract in reared spotted sand bass *Paralabrax maculatofasciatus* larvae. Aquaculture. 2003;219: 633–644.

[32] Lazo JP, Darias MJ, Gisbert E. Ontogeny of the digestive tract In: Holt GJ, editor. Larval Fish Nutrition. Oxford: John Wiley & Sons, Inc.; 2011 pp. 5–46.

[33] Douglas SE, Mandla S, Gallant JW. Molecular analysis of the amylase gene and its expression during development in the winter flounder, *Pleuronectes americanus* . Aquaculture. 2000;190: 247–260.

[34] Frøystad MK, Lilleeng E, Sundby A, Krogdahl Å. Cloning and characterization of a-amylase from Atlantic salmon (Salmo salar L.). Comp Biochem Physiol A Mol Integr Physiol. 2006;145: 479–492. 17020811 10.1016/j.cbpa.2006.08.003

[35] Krogdahl Å, Nordrum M, Sørensen M, Brudeseth L, Røsjø C. Effects of diet composition on apparent nutrient absorption along the intestinal tract and of subsequent fasting on mucosal disaccharidase activities and plasma nutrient concentration in Atlantic salmon *Salmo salar* L. Aquacult Nutr. 1999;5: 121–133.

[36] Einarsson S, Davies PS, Talbot C. Effect of exogenous cholecystokinin on the discharge of the gallbladder and the secretion of trypsin and chymotrypsin from the pancreas of the Atlantic salmon, *Salmo salar* L. Comp Biochem Physiol C Pharmacol Toxicol Endocrinol. 1997;117: 63–67. 9185328 10.1016/s0742-8413(96)00226-5

[37] Deng X, Guarita DR, Pedroso MRA, Kreiss C, Wood PG, Sved AF, et al PYY inhibits CCK-stimulated pancreatic secretion through the area postrema in unanesthetized rats. Am J Physiol—Reg Integrat Comp Physiol. 2001;281: R645–R653. 10.1152/ajpregu.2001.281.2.R645 11448870

[38] Murashita K, Fukada H, Hosokawa H, Masumoto T. Cholecystokinin and peptide Y in yellowtail (*Seriola quinqueradiata*): Molecular cloning, real-time quantitative RT-PCR, and response to feeding and fasting. Gen Comp Endocrinol. 2006;145: 287–297. 16242687 10.1016/j.ygcen.2005.09.008

[39] Murashita K, Kurokawa T, Nilsen TO, Rønnestad I. Ghrelin, cholecystokinin, and peptide YY in Atlantic salmon (*Salmo salar*): Molecular cloning and tissue expression. Gen Comp Endocrinol. 2009;160: 223–235. 10.1016/j.ygcen.2008.11.024 19073185

[40] Rønnestad I, Kamisaka Y, Conceição LEC, Morais S, Tonheim SK. Digestive physiology of marine fish larvae: Hormonal control and processing capacity for proteins, peptides and amino acids. Aquaculture. 2007;268: 82–97.

[41] Boulhic M, Gabaudan J. Histological study of the organogenesis of the digestive system and swim bladder of the Dover sole, *Solea solea* (Linnaeus 1758). Aquaculture. 1992;102: 373–396.

[42] Faulk CK, Benninghoff AD, Holt GJ. Ontogeny of the gastrointestinal tract and selected digestive enzymes in cobia *Rachycentron canadum* (L.). J Fish Biol. 2007;70: 567–583.

[43] García Hernández MP, Lozano MT, Elbal MT, Agulleiro B. Development of the digestive tract of sea bass (*Dicentrarchus labrax* L.). Light and electron microscopic studies. Anat Embryol. 2001;204: 39–57. 11506432 10.1007/s004290100173

[44] Govoni JJ, Boehlert GW, Watanabe Y. The physiology of digestion in fish larvae. Environ Biol Fish. 1986;16: 59–77.

[45] Pedersen T, Falk-Petersen IB. Morphological changes during metamorphosis in cod (*Gadus morhua* L), with particular reference to the development of the stomach and pyloric caeca. J Fish Biol. 1992;41: 449–461.

[46] Pradhan PK, Jena JK, Mitra G, Sood N, Gisbert E. Ontogeny of the digestive tract in butter catfish *Ompok bimaculatus* (Bloch) larvae. Fish Physiol Biochem. 2012;38: 1601–1617. 10.1007/s10695-012-9655-8 22585417

[47] Sarasquete MC, Polo A, Yufera M. Histology and histochemistry of the development of the digestive system of larval gilthead seabream, *Sparus aurata* L. Aquaculture. 1995;130: 79–92.

[48] Segner H, Storch V, Reinecke M, Kloas W, Hanke W. The development of functional digestive and metabolic organs in turbot, *Scophthalmus maximus* . Mar Biol. 1994;119: 471–486.

[49] Stroband HWJ, Kroon AG. The development of the stomach in *Clarias lazera* and the intestinal absorption of protein macromolecules. Cell Tis Res. 1981;215: 397–415. 10.1007/BF00239123 7214483

[50] Einarsdóttir IE, Power DM, Jönsson E, Björnsson BT. Occurrence of ghrelin-producing cells, the ghrelin receptor and Na+,K +-ATPase in tissues of Atlantic halibut (*Hippoglossus hippoglossus*) during early development. Cell Tis Res. 2011;344: 481–498. 10.1007/s00441-011-1158-x 21461677

[51] Kojima M, Kangawa K. Ghrelin: structure and function. Physiol Rev. 2005;85: 495–522. 15788704 10.1152/physrev.00012.2004

[52] Moen AGG, Murashita K, Finn RN. Ontogeny of energy homeostatic pathways via neuroendocrine signaling in Atlantic salmon. Dev Neurobiol. 2010;70: 649–658. 10.1002/dneu.20803 20506200

[53] Gawlicka A, Teh SJ, Hung SSO, Hinton DE, Noüe J. Histological and histochemical changes in the digestive tract of white sturgeon larvae during ontogeny. Fish Physiol Biochem. 1995;14: 357–371. 10.1007/BF00003374 24197527

[54] Darias MJ, Murray HM, Martínez-Rodríguez G, Cárdenas S, Yúfera M. Gene expression of pepsinogen during the larval development of red porgy (*Pagrus pagrus*). Aquaculture. 2005;248: 245–252.

[55] Darias MJ, Murray HM, Gallant JW, Douglas SE, Yúfera M, Martínez-Rodríguez G. Ontogeny of pepsinogen and gastric proton pump expression in red porgy (*Pagrus pagrus*): Determination of stomach functionality. Aquaculture. 2007;270: 369–378.

[56] Kurokawa T, Kagawa H, Ohta H, Tanaka H, Okuzawa K, Hirose K. Development of digestive organs and feeding ability in larvae of Japanese eel (*Anguilla japonica*). Can J Fish Aquat Sci. 1995;52: 1030–1036.

[57] Segner H, Rösch R, Verreth J, Witt U. Larval nutritional physiology: Studies with *Clarias gariepinus*, *Coregonus lavaretus* and *Scophthalmus maximus* . J World Aquacult Soc. 1993;24: 121–134.

[58] Buddington RK, Krogdahl Å. Hormonal regulation of the fish gastrointestinal tract. Comp Biochem Physiol A Mol Integr Physiol. 2004;139: 261–271. 15556381 10.1016/j.cbpb.2004.09.007

[59] Zambonino Infante JL, Cahu CL. Ontogeny of the gastrointestinal tract of marine fish larvae. Comp Biochem Physiol C Toxicol Pharmacol. 2001;130: 477–487. 11738635 10.1016/s1532-0456(01)00274-5

[60] Zhou J, Stubhaug I, Torstensen B. Trans-membrane uptake and intracellular metabolism of fatty acids in Atlantic salmon (*Salmo salar* L.) hepatocytes. Lipids. 2010;45: 301–311. 10.1007/s11745-010-3396-1 20186497

[61] Niot I, Poirier H, Tran TTT, Besnard P. Intestinal absorption of long-chain fatty acids: Evidence and uncertainties. Prog Lipid Res. 2009;48: 101–115. 19280719 10.1016/j.plipres.2009.01.001

[62] Gatlin DM, Barrows FT, Brown P, Dabrowski K, Gaylord TG, Hardy RW, et al Expanding the utilization of sustainable plant products in aquafeeds: a review. Aquacult Res. 2007;38: 551–579.

[63] He T, Xiao Z, Liu Q, Ma D, Xu S, Xiao Y, et al Ontogeny of the digestive tract and enzymes in rock bream *Oplegnathus fasciatus* (Temminck et Schlegel 1844) larvae. Fish Physiol Biochem. 2012;38: 297–308. 10.1007/s10695-011-9507-y 21604160

[64] Noaillac-Depeyre J, Gas N. Absorption of protein macromolecules by the enterocytes of the carp (*Cyprinus carpio* L.). Ultrastructural and cytochemical study. Zeitschr Zellforsch Mikroskop Anat. 1973;146: 525–541. 4361952 10.1007/BF02347181

[65] Gargiulo AM, Ceccarelli P, Dall'Aglio C, Pedini V. Histology and ultrastructure of the gut of the tilapia (*Tilapia* spp.), a hybrid teleost. Anat Histol Embryol. 1998;27: 89–94. 9591370 10.1111/j.1439-0264.1998.tb00162.x

[66] Ng AN, de Jong-Curtain TA, Mawdsley DJ, White SJ, Shin J, Appel B, et al Formation of the digestive system in zebrafish: III. Intestinal epithelium morphogenesis. Dev Biol. 2005;286: 114–135. 16125164 10.1016/j.ydbio.2005.07.013

[67] Wallace KN, Akhter S, Smith EM, Lorent K, Pack M. Intestinal growth and differentiation in zebrafish. Mech Develop. 2005;122: 157–173. 15652704 10.1016/j.mod.2004.10.009

[68] Tipsmark CK, Sørensen KJ, Madsen SS. Aquaporin expression dynamics in osmoregulatory tissues of Atlantic salmon during smoltification and seawater acclimation. J Exp Biol. 2010;213: 368–379. 10.1242/jeb.034785 20086120

[69] Heming TA, Buddington RK. Yolk absorption in embryonic and larval fishes In: Hoar WS, Randall DJ, editors. Fish Physiology Vol XI The Physiology of Developing Fish Part A Eggs and Larvae. San Diego: Academic Press, Inc.; 1988 pp. 407–446.

[70] Gu J, Bakke AM, Valen EC, Lein I, Krogdahl Å. *Bt*-maize (MON810) and non-GM soybean meal in diets for Atlantic salmon (*Salmo salar* L.) juveniles—impact on survival, growth performance, development, digestive function, and transcriptional expression of intestinal immune and stress responses. PloS ONE. 2014;9: e99932 10.1371/journal.pone.0099932 24923786 PMC4055707

[71] Bakke-McKellep AM, Frøystad MK, Lilleeng E, Dapra F, Refstie S, Krogdahl Å, et al Response to soy: T-cell-like reactivity in the intestine of Atlantic salmon, *Salmo salar* L. J Fish Dis. 2007;30: 13–25. 17241401 10.1111/j.1365-2761.2007.00769.x

[72] Krogdahl Å, Bakke-McKellep AM, Røed KH, Baeverfjord G. Feeding Atlantic salmon *Salmo salar* L. soybean products: effects on disease resistance (furunculosis), and lysozyme and IgM levels in the intestinal mucosa. Aquacult Nutr. 2000;6: 77–84.

[73] Sahlmann C, Sutherland BJGG, Kortner TM, Koop BF, Krogdahl Å, Bakke AM. Early response of gene expression in the distal intestine of Atlantic salmon (*Salmo salar* L.) during the development of soybean meal induced enteritis. Fish Shellfish Immunol. 2012;34: 599–609. 10.1016/j.fsi.2012.11.031 23246810

[74] Skugor S, Grisdale-Helland B, Refstie S, Afanasyev S, Vielma J, Krasnov A. Gene expression responses to restricted feeding and extracted soybean meal in Atlantic salmon (*Salmo salar* L.). Aquacult Nutr. 2011;17: 505–517.

[75] Chikwati EM, Sahlmann C, Holm H, Penn H, Krogdahl A, Bakke AM. Alterations in digestive enzyme activities during the development of diet-induced enteritis in Atlantic salmon, *Salmo salar* L. Aquaculture. 2013;402: 28–37.

